# Preliminary Analysis of Intestinal Microbiota in Golden Retrievers Prone to Dilated Cardiomyopathy Due to Taurine Deficiency

**DOI:** 10.3390/vetsci12121120

**Published:** 2025-11-26

**Authors:** Matilda Rachele Dametti, Mara Bagardi, Sara Ghilardi, Giulietta Minozzi, Michele Polli, Paola Giuseppina Brambilla, Eleonora Fusi

**Affiliations:** 1 Department of Veterinary Medicine and Animal Science-DIVAS, University of Milan, Via dell’Università 6, 26900 Lodi, Italy; mara.bagardi@unimi.it (M.B.); sara.ghilardi@guest.unimi.it (S.G.); giulietta.minozzi@unimi.it (G.M.); michele.polli@unimi.it (M.P.); paola.brambilla@unimi.it (P.G.B.); 2 Anicura Clinica Veterinaria Malpensa, Via G. Marconi N. 27, 21017 Samarate, VA, Italy

**Keywords:** nutritional dilated cardiomyopathy, taurine, golden retriever, microbiota

## Abstract

Taurine plays a key role in cardiovascular and gastrointestinal health in dogs and may be deficient in some predisposed breeds despite consuming complete petfood. This study aimed to explore potential associations between serum taurine concentration and gut microbiota composition in clinically healthy Golden Retrievers, a breed predisposed to taurine deficiency-associated dilated cardiomyopathy. Eleven adult dogs, which were fed the same extruded commercial diet and living in the same kennel, underwent a nutritional evaluation, fecal analysis, and measurement of serum concentrations of taurine, folates, and vitamin B12. Taurine levels were below reference values in eight out of eleven dogs, and 50% of the tested dogs showed reduced serum folate. Microbiota analysis based on 16S rRNA sequencing revealed subtle compositional shifts in dogs with lower taurine levels, including a depletion of mucus-associated *Deferribacterota* and an enrichment of bile-sensitive taxa such as Lactobacillus and Streptococcus. While alpha and beta diversity metrics did not differ significantly between groups, unweighted UniFrac indicated a trend toward separation. These findings suggest that hypotaurinemia in Golden Retrievers may be associated with specific alterations in the gut microbiota, potentially reflecting subclinical dysbiosis. Further studies integrating metabolomic profiling are needed to clarify the functional implications of these microbial changes on taurine metabolism.

## Simple Summary

Taurine is an important nutrient for dogs, particularly for their heart and gut health. Some Golden Retrievers may develop low taurine levels even when eating a balanced commercial diet. This can potentially increase their risk for heart disease, such as dilated cardiomyopathy. At the same time, bacteria in the gut—known as the intestinal microbiota—may play a role in taurine metabolism and absorption. In this study, we evaluated serum taurine concentrations and intestinal microbiota in eleven healthy Golden Retrievers, all eating the same diet. We found that most of these dogs had low taurine levels, and some also showed reduced concentrations of folates, a vitamin linked to gut function. Interestingly, dogs with lower taurine levels showed subtle changes in their gut microbiota, particularly in bacteria known to interact with bile and gut health. These results suggest that imbalances in gut bacteria might influence taurine levels in dogs, even in the absence of visible illness.

## 1. Introduction

Taurine plays a crucial role in maintaining animal health, with significant implications for canine cardiovascular and gastrointestinal systems.

Taurine, a sulfur-containing amino acid, is conditionally essential in dogs, meaning that it can be synthesized endogenously or obtained through diet, but its levels may become insufficient under certain conditions. This deficiency is particularly concerning in predisposed breeds, such as the Golden Retriever, which is prone to developing diet-associated dilated cardiomyopathy (DCM).

Dogs require a balanced intake of macronutrients, proteins, lipids, and carbohydrates, as well as micronutrients, such as vitamins and minerals, for optimal health. Although taurine is categorized as non-essential in many species, in dogs it is better described as conditionally essential, since endogenous synthesis from precursors like methionine and cysteine is often insufficient to meet physiological needs [1]. This taurine shortfall may stem from metabolic inefficiencies and sometimes by dietary deficiencies [2]. Indeed, taurine is predominantly sourced from animal-based products, such as meat, fish, and their derivatives, which are naturally rich in this amino acid [3].

Taurine is involved in a wide range of physiological processes. It functions as a neurotransmitter, immunomodulator, antioxidant, and cytoprotective agent. Additionally, taurine plays an important role in gene expression and in energy metabolism by promoting the oxidative metabolism of fatty acids [4]. Furthermore, it also contributes to bile acid conjugation, which is essential for lipid digestion and absorption [5]. Importantly, taurine supports cardiovascular health by helping to stabilize cell membranes, regulate calcium transport, and reduce oxidative and inflammatory stress on cardiomyocytes [6]. Consequently, taurine deficiency has been linked to various metabolic and cardiovascular disorders in dogs.

Among these, DCM is a well-characterized and potentially life-threatening myocardial condition marked by the dilation of cardiac chambers and decreased myocardial contractility, ultimately leading to congestive heart failure [7]. While DCM may have multifactorial etiologies, including genetic predisposition, inflammation, and autoimmune mechanisms, nutritional deficiencies, particularly of taurine and carnitine, have been implicated as risk factors [8]. In particular, taurine deficiency and subsequent hypotaurinemia have been associated with the consumption of so-called “Boutique, Exotic, and Grain-Free” (BEG) diets, especially those rich in legumes, which may lack adequate levels of methionine and cysteine or interfere with their gastrointestinal availability [9,10]. In predisposed breeds like Golden Retrievers, these diets have been linked to decreased serum taurine levels and an increased risk of developing DCM [8].

Beyond its direct nutritional role, taurine metabolism appears to be influenced by the gut microbiota, a complex community of microorganisms that inhabit the gastrointestinal tract.

These microbes perform essential functions such as the fermentation of complex carbohydrates, synthesis of short-chain fatty acids, and production of vitamins and other bioactive compounds [11,12]. In the context of taurine metabolism, certain bacterial species express enzymes capable of deconjugating taurine from bile salts, thereby influencing its bioavailability and systemic absorption [13]. Hence, alterations in microbial composition, often described as dysbiosis, may have a direct impact on taurine homeostasis.

Dysbiosis, characterized by changes in the diversity or abundance of microbial taxa, can impair nutrient absorption, disrupt intestinal barrier integrity, and influence systemic inflammation [14]. Notably, growing evidence supports a connection between gut microbiota composition and cardiovascular disease in humans and animals alike [15]. The so-called gut–heart axis reflects this relationship, whereby microbial metabolites such as trimethylamine N-oxide (TMAO) and short-chain fatty acids can influence cardiac function and disease progression. Specifically, reduced butyrate production and elevated concentrations of TMAO have been linked to conditions such as hypertrophic cardiomyopathy, atherosclerosis, and congestive heart failure [16,17].

Despite increasing recognition of the importance of the gut–heart axis, the interplay between diet, microbiota, and taurine metabolism in dogs, particularly those predisposed to nutritional DCM, remains poorly understood. Investigating these mechanisms in healthy animals from a predisposed breed and under controlled nutritional and environmental conditions represents an essential step for identifying early biological features potentially linked to disease risk.

Therefore, the present study was conducted as a cross-sectional observational exploratory pilot investigation aimed at characterizing the gut microbiota of clinically healthy Golden Retrievers previously confirmed to be free of cardiac abnormalities, and at identifying microbial compositional differences between individuals with normal taurine concentrations and those with hypotaurinemia.

## 2. Materials and Methods

### 2.1. Animals and Diet

This cross-sectional observational exploratory study is part of a broader research project involving 48 Golden Retrievers, a breed selected for its known predisposition to clinically manifest taurine deficiency. The 11 dogs were enrolled through the cardiology and clinical nutrition services of the Veterinary Teaching Hospital of the University of Milan. All dogs were deemed clinically healthy following a comprehensive physical examination and were up to date with vaccinations and regularly treated with preventatives for heartworm and gastrointestinal parasites. The study protocol was approved by the Animal Welfare Organisation of the Università degli Studi di Milano (OPBA_105_2023). Data regarding CBC, serum biochemistry, comprehensive thyroid panel, troponin I concentration, urinalysis, and echocardiographic findings were previously reported and confirmed the absence of clinical hematological and structural or functional cardiac abnormalities at the time of sampling [18].

The dogs were housed in a private facility located in Lombardy, Italy. The kennel was situated in a temperate climate area of Northern Italy. Each dog was housed individually in an indoor box with access to an outdoor run and participated in three daily exercise sessions in a shared paddock with conspecifics. Dogs did not engage in structured physical or sporting activities beyond these walks. To minimize dietary and environmental influences on microbiome composition, only dogs fed the same extruded commercial dry petfood and living in the same kennel were included in the study. No changes or adaptations to the diet were introduced before or during the study period, as all dogs had been consistently fed the same commercial food prior to enrollment.

The formulation contained a single protein source, pork, provided as dehydrated protein; hydrolyzed liver; and lard as the fat source, along with a single carbohydrate source, rice, which was whole and flaked. Additional ingredients included dehydrated beet pulp, brewer’s yeast, fructooligosaccharides, nutritional additives (minerals and vitamins), and a flavoring agent (chestnut extract). The chemical composition of the diet is detailed in Table 1.

The metabolizable energy of the diet was calculated using the formulas outlined in the FEDIAF guidelines (2024) [19].

Each dog was fed twice daily with a total amount of kibble sufficient to meet its individual daily energy requirements, calculated using the formula recommended by the FEDIAF guidelines:

Kcal/day = 110 × Ideal Body Weight (kg)^0.75^. (1)

Additionally, dogs had unrestricted access to fresh drinking water.

The final group of dogs comprised 10 intact females (none pregnant at the time of sampling) and 1 intact male, aged between 1 and 9 years, with an average age of 3.7 years.

Given these strict enrollment criteria, the study was designed as a preliminary exploratory pilot investigation to provide initial data under highly controlled conditions.

### 2.2. Nutritional Assessment

Nutritional status of all subjects was evaluated according to Nutritional Assessment Guidelines for Dogs and Cats [20]. Body weight (kg), using a large pet scale, Body Condition Score (BCS) on a 1 to 9 scale, and Muscle Condition Score (MCS) on a 1 to 4 scale were recorded. Measurements were carried out on the same day as sample collection, using the same calibrated veterinary scale to ensure consistency. Each assessment was performed by the same experienced veterinarian (DVM), minimizing inter-operator variability.

### 2.3. Taurine, Folates and B12 Serum Concentration Measurement

Serum samples were collected, centrifuged immediately after harvest, and stored at −80 °C until shipment to the reference laboratory to preserve analyte stability.

Serum taurine concentrations were measured using a liquid chromatography–tandem mass spectrometry (LC-MS/MS) method developed and validated by the San Marco Veterinary Laboratory. As no established reference range exists for serum taurine, we compared our results to the range of 110–272 nmol/mL reported by Kathrani et al. (2018) in healthy dogs [21].

To investigate potential gastrointestinal malabsorption, serum concentrations of folates and vitamin B12 were analyzed at MyLav Laboratory.

### 2.4. Fecal Evaluation

Stool samples were collected during the evaluation and subjected to a visual assessment of their appearance by the same clinician (DVM).

Consistency was evaluated using the Purina Fecal Scoring Chart, a canine-adapted version of the original Bristol Stool Chart, on a 1 to 7 scale [22]. The color of the fecal samples was also assessed to detect potential abnormalities, including green, red, orange, yellow, white, black, or gray discolorations.

In addition, specimens were examined for the presence of visible abnormalities, such as mucus, blood, pus, parasites, undigested food residues or foreign material.

### 2.5. Microbiota Analysis

For microbiota analysis, a fecal swab (FecalSwab™, Copan Diagnostics Inc., Murrieta, CA, USA) was collected directly from the same stool sample during the evaluation, carefully avoiding contamination from external surfaces. Once collected, samples were placed into the commercial kit tube, and shipped at room temperature according to the manufacturer’s instructions to the reference laboratory, ensuring preservation of microbial DNA integrity. The swabbed samples were then shipped to the Laboratory of Microbial Ecology and Genomics at the Istituto Zooprofilattico Sperimentale delle Venezie (Legnaro, Padua).

Cell lysis was performed using a combination of chemical and mechanical methods (QIAamp PowerFecal DNA Kit, QIAGEN), starting with 250 μL of diluted fecal sample. Total DNA was extracted from 200 μL of the lysate using the Cador Pathogen 96 QIAcube HT Kit (QIAGEN). The extracted DNA was resuspended in 100 μL of nuclease-free water and stored at −80 °C until preparation for sequencing.

The 16S rRNA gene was amplified using a standard protocol with modified primers [23]. In brief, the PCR conditions were as follows: initial denaturation at 94 °C for 1 min, followed by 25 amplification cycles with denaturation at 94 °C for 30 s, annealing at 55 °C for 30 s, and extension at 68 °C for 45 s. After the cycles, a final extension was carried out at 68 °C for 7 min. The primers used were specific for the V3–V4 region of the 16S rRNA gene:

Pro341f: 5′-TCGTCGGCAGCGTCAGATGTGTATAAGAGACAGCCTACGGGNBGCASCAG-3′

Pro805R: 5′-GTCTCGTGGGCTCGGAGATGTGTATAAGAGACAGCAGGACTACNVGGGTATCTAATCC-3′

The amplicons were purified using Agencourt XP magnetic beads (Beckman Coulter, Brea, CA, USA) and then amplified using the HiSeq platform with the Nextera XT Index Kit (Illumina, San Diego, CA, USA). All amplified sequences were normalized using SequalPrep (Thermo Fisher, Waltham, MA, USA) and precipitated with magnetic beads (Agencourt XP 0.8×). The libraries were sequenced on a MiSeq platform (Illumina, San Diego, CA, USA) using the V3 kit 300PE. Taxonomic classification of all reads was performed using the QIIME2 platform and the SILVA reference database (version 138), assigning sequences to the lowest possible taxonomic rank [24]. Sequence quality was evaluated using FastQC, and reads with a Phred quality score below 30 were discarded. Good’s coverage values above 0.98 were considered indicative of adequate sequencing depth.

### 2.6. Statistical Analyses

Data from the nutritional assessment and fecal evaluation were analyzed using descriptive statistics. Relationships among serum taurine, folate, and vitamin B12 concentrations; nutritional parameters (body weight, BCS, and MCS); fecal scores (consistency and color); and microbial diversity indices (alpha and beta) were explored using simple linear regression and Pearson correlation tests, performed with GraphPad Prism v.10.3.1 (GraphPad Software, Boston, MA, USA).

Microbiota sequencing data were processed using the QIIME2 pipeline (version 2022.8) [25]. Primer sequences were trimmed with the cutadapt plugin, while quality filtering, and chimera removal were carried out using DADA2 [26,27]. To normalize sequencing depth, samples were rarefied to 13,500 reads.

Alpha diversity was evaluated using the Chao1, Shannon and Pielou indexes. Beta diversity was assessed using Bray–Curtis dissimilarity and both weighted and unweighted UniFrac distances, and graphically represented through Principal Coordinates Analysis (PCoA). Group-wise differences in beta diversity were assessed using Analysis of Similarities (ANOSIM) and Permutational Multivariate Analysis of Variance (PERMANOVA). The R value generated by ANOSIM is scaled between −1.0 and 1.0. An ANOSIM R value close to 1.0 suggests dissimilarity between groups, and an R value close to 0 suggests similarity between groups. Taxonomic composition was explored through differential abundance analysis using Analysis of Compositions of Microbiomes (ANCOM) and Analysis of Compositions of Microbiomes with Bias Correction (ANCOM-BC) frameworks [28,29].

Data are presented as mean ± standard deviation. A threshold of *p*  <  0.05 was considered statistically significant, with correction for multiple comparisons performed using the Benjamini–Hochberg False Discovery Rate (FDR) adjustment.

## 3. Results

### 3.1. Nutritional Assessment

The body weights of the 11 recruited subjects are reported in Table 2. Mean weight was 26.5 ± 3.95 kg, ranging from 21 to 33.5 kg. Body Condition Score (BCS) assessment revealed a generalized condition of overweight, with 73% of the dogs scoring 6 and one dog scoring 7/9. The remaining 18% of the subjects had a BCS of 5/9 (5.91 ± 0.54).

The Muscle Condition Score (MCS) was found to be adequate in all examined subjects (4 ± 0).

### 3.2. Taurine, Folates and B12 Serum Concentration Measurement

Serum taurine concentrations averaged 93.7 ± 58.8 nmol/mL, ranging from 31.9 to 196.1 nmol/mL. Individual serum taurine concentrations for each dog are detailed in Table 3, of which 8 out of the 11 dogs had values below the 110–272 nmol/mL range selected from the literature.

**Figure 1:**
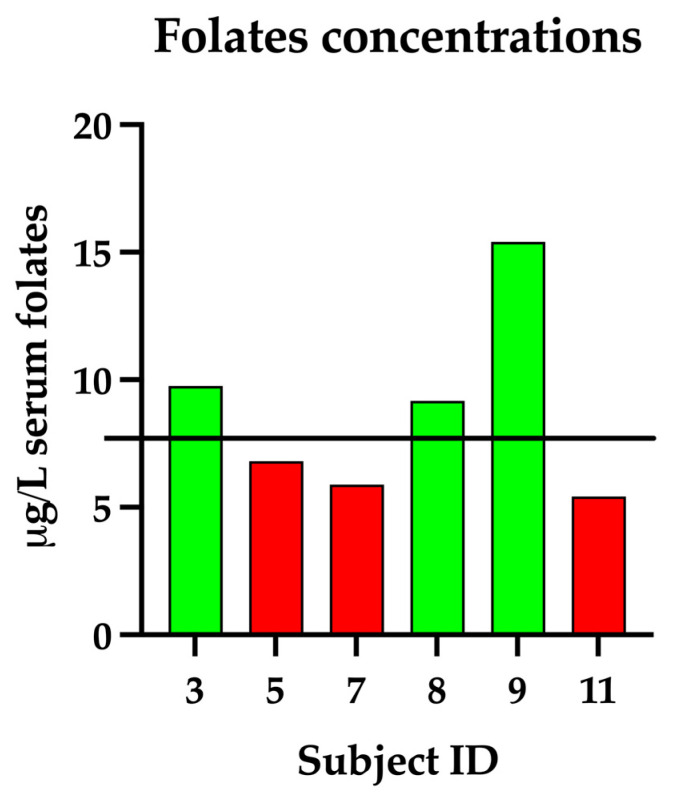
A bar graph showing individual serum concentrations of folate (µg/L) for the enrolled dogs. The black horizontal line represents the lower threshold of the laboratory’s reference range (7.7 µg/L). Bars falling below the reference threshold are colored red, while values within the normal range are colored green.

In addition to taurine, the evaluation of other micronutrients relevant to gastrointestinal and metabolic health was conducted. Due to limited serum availability, folate concentrations were assessed in only six dogs, while vitamin B12 levels were measured in seven. The mean serum folate concentration was 8.74 ± 3.70 µg/L, ranging from 5.43 to 15.4 µg/L. Individual serum folate concentrations for the assessed dogs are graphically presented in Figure 1. Of the six dogs tested, three (50%) exhibited folate concentrations below the laboratory’s reference interval (7.7–24 µg/L).

**Figure 2:**
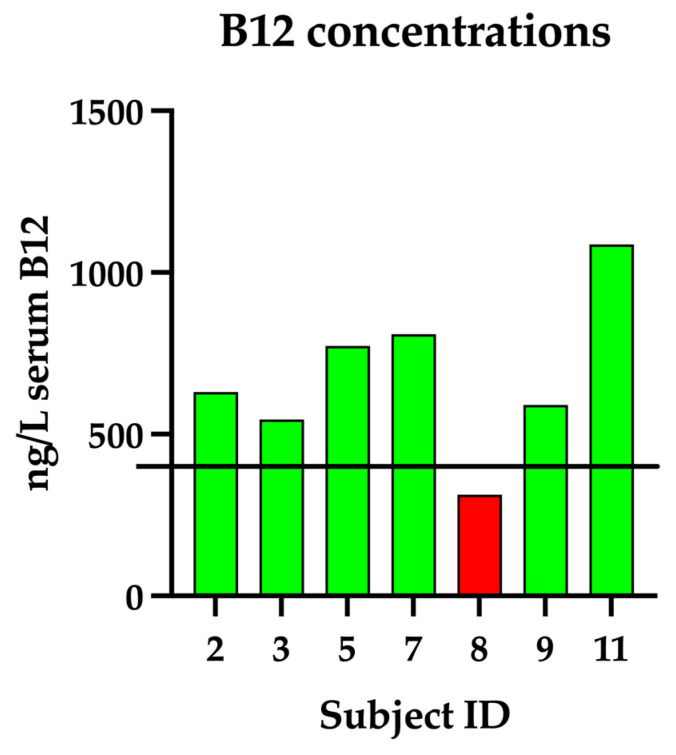
A bar graph showing individual serum concentrations of B12 (ng/L) for the enrolled dogs. The black horizontal line is set at 400 ng/L, indicating the suggested threshold below which supplementation is recommended. Bars below this value are colored red, those above are colored green.

In contrast, serum vitamin B12 concentrations averaged 678.43 ± 242.92 ng/L, ranging from 313 to 1087 ng/L. Individual serum vitamin B12 concentrations are illustrated in Figure 2. While all measurements fell within the laboratory’s established reference range (251–908 ng/L), emerging evidence suggests that serum B12 concentrations below 400 ng/L may be suboptimal and warrant supplementation, either orally or, in more severe cases, via subcutaneous injections [30]. Based on this threshold, one dog in the study, with a B12 level of 313 ng/L, would be considered deficient and in need of supplementation.

Correlation analysis did not reveal any significant associations among serum taurine, folate, and vitamin B12 concentrations or between these biochemical parameters and body weight, BCS, or MCS.

### 3.3. Fecal Evaluation

Most fecal samples displayed an adequate consistency, as indicated by their fecal scores (4.36 ± 0.67). Specifically, 73% of the samples were assigned a score of 4/7. Two subjects exhibited a slight decrease in stool consistency, corresponding to a score of 5/7, while one dog received a score of 6/7.

All samples displayed a dark brown coloration, except for one subject, which produced lighter-colored fecal matter.

No stool specimen showed the presence of foreign material.

Individual data on fecal consistency, color, and presence of foreign material are presented in Table 4.

No significant correlations were observed between fecal consistency or color and serum taurine, folate, or vitamin B12 concentrations, nor with alpha or beta diversity indices.

### 3.4. Microbiota Analysis

**Figure 3:**
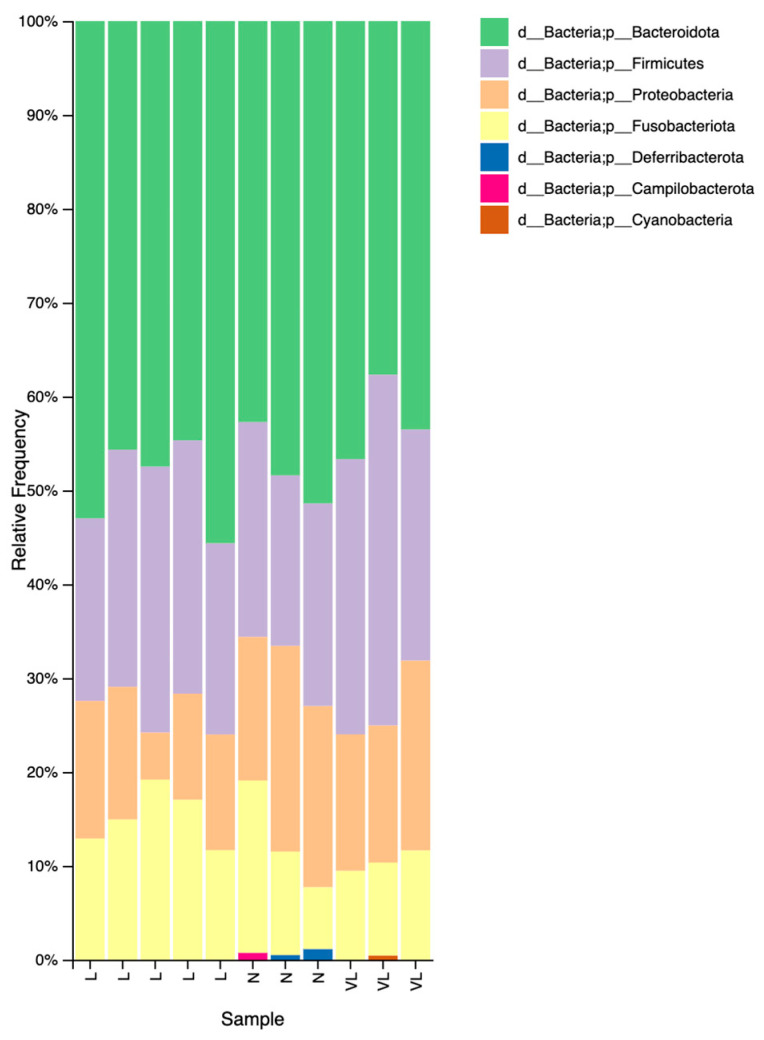
Taxa at phylum level relative frequencies’ barplot.

To allow comparison, dogs were assigned to three groups based on serum taurine concentrations: Normal (N) (155.1–200 nmol/mL), Low (L) (70.1–155 nmol/mL), and Very Low (VL) (0–70 nmol/mL). A graphical representation of the relative abundance of bacterial taxa at the phylum level across taurine groups is shown in Figure 3.

In the N group, the predominant bacterial phylum was *Bacteroidota* (median relative abundance: 48.41%; range: 42.73–51.40%), followed by *Firmicutes* (median relative abundance: 21.57%, range: 18.16–22.87%), *Proteobacteria* (median relative abundance: 19.32%; range: 15.30–21.93%), *Fusobacteriota* (median relative abundance: 11.02%; range: 6.59–18.36%), *Deferribacterota* (median relative abundance: 0.49%; range: 0–1.13%), and *Campylobacterota* (median relative abundance: 0%; range: 0–0.73%). A total of 44 bacterial species were identified in this group.

In the L group, *Bacteroidota* remained the dominant phylum (median relative abundance: 47.48%; range: 44.67–55.64%), followed by *Firmicutes* (median relative abundance: 25.25%; range: 19.44–28.31%) and *Fusobacteriota* (median relative abundance: 14.93%; range: 11.67–19.18%), which showed a higher relative abundance than *Proteobacteria* (median relative abundance: 12.32%; range: 5.03–14.67%). No members of the *Deferribacterota* or *Campylobacterota* phyla were detected. This group exhibited the highest species richness, with a total of 62 species identified.

In the VL group, the microbial composition was also dominated by *Bacteroidota* (median relative abundance: 43.53%; range: 37.68–46.67%), followed by *Firmicutes* (median relative abundance: 29.33%; range: 24.60–37.36%), *Proteobacteria* (median relative abundance: 14.62%; range: 14.54–20.24%), and *Fusobacteriota* (median relative abundance: 9.90%; range: 9.46–11.63%). *Deferribacterota* and *Campylobacterota* were not detected, but low levels of *Cyanobacteria* were observed (median relative abundance: 0%; range: 0–0.44%). A total of 50 bacterial species were identified in this group.

Focusing on alpha diversity, no statistically significant differences were observed among the groups for any of the evaluated indices, including Chao1, Shannon, and Pielou evenness.

**Figure 4:**
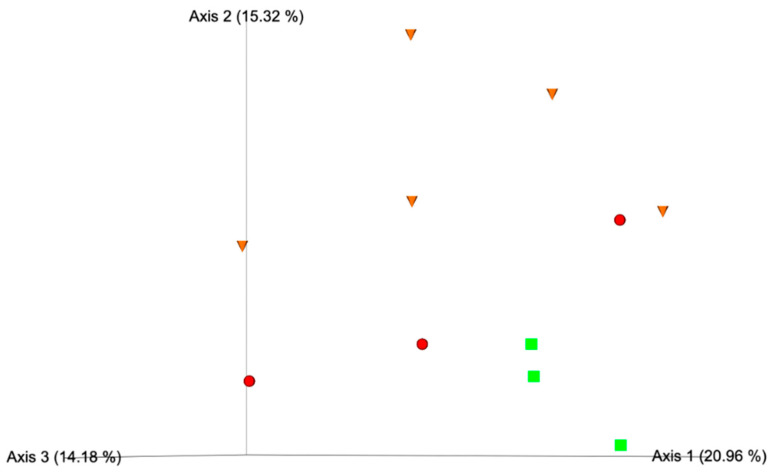
Three-dimensional Principal Coordinates Analysis (PCoA) plot based on Unweighted UniFrac distances. Taurine groups are distinguished by both color and shape: N (green squares), L (orange triangles), and VL (red spheres).

In terms of beta diversity, Principal Coordinates Analysis (PCoA) did not reveal clear clustering of samples according to taurine groups. Additionally, no statistically significant differences were observed across groups when comparing Bray–Curtis, Unweighted UniFrac, or Weighted UniFrac distances using ANOSIM. An exception was noted for the Unweighted UniFrac metric, which showed a significant difference between the N and L groups (*p* = 0.02); however, it did not survive FDR correction (q = 0.069), though still indicating a trend toward separation. The 3D PCoA plot based on Unweighted UniFrac distances is presented in Figure 4.

Regarding taxonomic differences, a reduction in the phylum *Deferribacterota* was observed in both the L and VL groups compared to the N group (L: *p* = 0.004, q = 0.02; V L: *p* = 0.003, q = 0.01).

At the class level, the VL group exhibited a significant increase in *Firmicutes Bacilli* (*p* < 0.001, q < 0.001) and a marked depletion in *Firmicutes Negativicutes* (*p* < 0.001, q = 0.001). *Deferribacterota Deferribacteres* was reduced in both the L (*p* = 0.005, q = 0.03) and VL (*p* = 0.007, q = 0.03) groups. A trend toward diminished abundance of *Proteobacteria Gammaproteobacteria* was also observed in the L group (*p* = 0.009, q = 0.055).

In the VL taurine group, the *Firmicutes Bacilli Lactobacillales* order was significantly enriched (*p* < 0.001, q < 0.001) compared to the N group.

At the family level, both *Lactobacillaceae* and *Streptococcaceae* (within *Firmicutes Bacilli Lactobacillales*) were increased in the VL group (*p* < 0.001, q < 0.001 for both).

Moreover, a significant enrichment of *Lactobacillus* and *Streptococcus* genera (familiae *Lactobacillaceae* and *Streptococcaceae*, respectively) was also observed in the VL group (*p* < 0.001, q < 0.001 for both). Additionally, the genus *UCG-004* (*Firmicutes Bacilli Erysipelotrichales Erysipelatoclostridiaceae*) was elevated in the L group compared to the Normal group (*p* < 0.001, q < 0.001).

## 4. Discussion

When evaluating nutritional status, the high prevalence of overweight individuals observed in this study, despite the absence of typical environmental risk factors for obesity, such as free-choice feeding, sedentary lifestyle, indoor confinement, or neutering, may be attributable to a breed-specific predisposition to excessive weight gain, as previously documented in the literature [31,32,33].

Adequate MCS, despite increased body fat, suggests that the subjects maintained sufficient levels of physical activity and protein intake to preserve lean body mass. Moreover, the absence of muscle wasting supports the assumption that none of the dogs were affected by chronic systemic disease or age-related sarcopenia, factors known to compromise muscle condition in canine patients [34].

Taurine is a conditionally essential amino acid whose circulating concentrations can vary widely depending on factors such as breed, dietary protein source and digestibility, sulfur amino acid content, and feeding frequency—with ad libitum feeding known to increase plasma levels [2,35]. As no universally accepted reference range for serum taurine is available, we referred to the values reported by Kathrani et al. (2018) in healthy dogs [21]. This range was adopted to ensure consistency with our previous manuscript reporting echocardiographic data from the same cohort, and because it was established using HPLC, a methodology more comparable to our LC–MS/MS than other techniques (e.g., ion-exchange chromatography with post-column ninhydrin derivatization). Moreover, deriving from a European population, it was considered more representative of our cohort than U.S.-based references. Nevertheless, other studies have proposed different ranges, from 44 to 224 nmol/mL in healthy dogs to 25–229 nmol/mL in acquired valvular disease and 1–247 nmol/mL in dilated or hypertrophic cardiomyopathy [36]. Given such variability, applying any single reference range may only partially interpret our data and represents a study limitation.

Based on the selected reference range, the majority of the enrolled dogs were found to be hypotaurinemic, with three subjects showing markedly reduced taurine concentrations and five others falling below the threshold. This finding could suggest cardiovascular implications, as taurine deficiency is linked to morphological heart changes, such as reduced ventricular wall thickness, decreased left ventricular mass, and diminished ventricular volume, and is associated with the development of dilated cardiomyopathy. Feeding dogs a diet that met AAFCO minimum nutrient requirements but was low in overall protein resulted in an approximate 50 nmol/mL decrease in plasma taurine concentrations, accompanied by echocardiographic findings consistent with dilated cardiomyopathy. These cardiac abnormalities improved with taurine supplementation and did not appear in dogs fed a low-fat diet enriched with carnitine, reinforcing the connection between dietary composition, taurine deficiency, and the development of cardiomyopathy [37].

In the present study, all dogs were fed the same commercial dry petfood, which included rice among its main ingredients. Notably, rice and other sources of fermentable fibers have been associated with reduced plasma concentrations of methionine and cystine, precursors essential for endogenous taurine synthesis [2]. Additionally, such ingredients may reduce the bioavailability of sulfur-containing amino acids and increase fecal losses, potentially contributing to taurine depletion [35].

Focusing on folic acid (Vitamin B9, folate) and vitamin B12 (cobalamin), these are water-soluble vitamins abundantly present in most commercial pet foods; therefore, dietary deficiencies are uncommon.

Folate (vitamin B9) and vitamin B12 (cobalamin) play essential roles in DNA synthesis, amino acid metabolism, and cellular function, particularly within rapidly renewing tissues such as those in the gastrointestinal epithelium [38]. Particularly, hypofolatemia, but also hypocobalaminemia in dogs have been associated with villous atrophy and biochemical abnormalities in brush borders, lysosomes, and the endoplasmic reticulum, when subjected to jejunal biopsies [39]. Moreover, the absorption and metabolism of these vitamins are intricately linked to gastrointestinal function and the gut microbiota. While hypocobalaminemia is a recognized marker of chronic enteropathies and malabsorption, emerging evidence suggests that hypofolatemia may also serve as a potential indicator of chronic intestinal dysfunction or dysbiosis, even when occurring in the absence of noticeable clinical signs [40]. Particularly, in presence of small intestine alterations, folates absorption has been found to be impaired due to a polyglutamate hydrolysis reduced activity and a damage to the mucosal carriers [41]. Moreover, folate levels can be significantly influenced by dysbiosis, as certain bacterial taxa—such as *Bifidobacterium* and *Lactobacillus spp.*—are capable of synthesizing folate, while others utilize it as a metabolic substrate. Consequently, bacterial overgrowth may lead to either an increase or a marked depletion in circulating folate concentrations [42,43]. The finding that 50% of the tested dogs displayed folate concentrations below the reference interval, despite normal serum cobalamin levels for most of the dogs, and the absence of gastrointestinal signs, may thus reflect subclinical alterations in gut function or microbial composition.

The predominantly normal fecal consistency and appearance among the enrolled dogs suggest overall gastrointestinal wellness and compatibility with the provided diet. A fecal score of 4/7 is considered ideal, reflecting proper gastrointestinal transit, hydration, and digestive efficiency [44,45]. Although the presence of softer stools in three dogs was not suggestive of overt clinical disease, it may point to mild gastrointestinal imbalances. Notably, one of these dogs also exhibited the lowest serum B12 concentration (313 ng/L), falling below the threshold of 400 ng/L at which cobalamin supplementation is generally recommended.

In the microbiota analysis, alpha and beta diversity analyses did not reveal marked alterations associated with taurine levels. Alpha diversity, assessed through multiple indices (Chao1, Shannon, and Pielou’s evenness), showed no statistically significant differences across the groups, suggesting that the overall richness and evenness of microbial communities were preserved despite variations in taurine status. This finding may indicate that taurine deficiency does not result in global microbial depletion or overgrowth but rather in more specific compositional shifts. Similar observations have been reported in experimental models, where dietary taurine supplementation or deficiency altered gut microbial composition without significantly affecting alpha diversity metrics [46]. In terms of beta diversity, no clear clustering of samples was observed by taurine status, and most distance metrics did not yield statistically significant differences. However, a trend toward phylogenetic separation between the N and L groups emerged using the Unweighted UniFrac metric, suggesting early shifts in specific, low-abundance lineages without disrupting the predominant community structure. Unweighted UniFrac is a presence–absence-based phylogenetic metric that is particularly sensitive to changes in rare or phylogenetically distinct taxa, unlike abundance-weighted measures such as Bray–Curtis or Weighted UniFrac [47]. Therefore, while global beta diversity appears comparable across taurine groups, this subtle Unweighted UniFrac finding could reflect incipient shifts within the low-abundance microbial community of the gut microbiome.

Focusing on taxonomic differences, a significant reduction in both *Deferribacterota* (phylum) and *Deferribacteres* (class) was observed in dogs with L and VL taurine status. Although typically low in abundance in the canine gut microbiome, this phylum, primarily represented by *Mucispirillum schaedleri* in the mammalian gut, plays a meaningful role in mucosal health [48]. In rodent models, *M. schaedleri* supports epithelial barrier stability, modulates local oxygen tension, and enhances colonization resistance and local immune response to pathogen invasion [49]. Similar reductions in this taxon have been reported in dogs with clinical leishmaniasis, when compared to asymptomatic and healthy counterparts [50]. The observed depletion in this study could reflect subtle disruptions in gut homeostasis due to taurine deficiency. In addition to its role in bile acid conjugation, taurine supports mucosal integrity by enhancing tight junction expression, stimulating goblet cell activity, and modulating inflammatory signaling, functions essential for maintaining the epithelial barrier and mucus layer [51,52]. Disruption of these processes could create a microenvironment less supportive of mucus-associated taxa such as *Deferribacterota*. Conversely, a reduction in this phylum could itself compromise mucosal homeostasis, as members like *Mucispirillum* contribute to maintaining the epithelial environment by scavenging oxygen and reactive species and supporting mucus production, activities that, when impaired, may indirectly hinder taurine uptake [53,54]. This potential bidirectional relationship suggests that taurine deficiency and mucus-associated microbiota loss may reinforce one another, contributing to a feedback loop that exacerbates both microbial imbalance and nutrient insufficiency.

In the VL group, a marked enrichment in *Firmicutes Bacilli Lactobacillales*, particularly *Lactobacillus* and *Streptococcus* genera was observed. In dogs, *Lactobacillus* species are saccharolytic microbes that ferment carbohydrates into short-chain fatty acids, particularly acetate and lactate, contributing to colonocyte energy supply and exerting local anti-inflammatory effects [55]. However, despite their probiotic reputation, *Lactobacillus* populations have been shown to increase under conditions of mucosal stress and altered bile acid metabolism [56,57]. Notably, this genus expresses bile salt hydrolase (BSH), an enzyme that catalyzes the deconjugation of bile acids from taurine or glycine, thereby participating in bile acid recycling and indirectly influencing taurine availability [58]. Nevertheless, *Lactobacillus* overgrowth has been associated with gastrointestinal pathologies in dogs, including chronic enteropathies and exocrine pancreatic insufficiency, where it contributes to fecal accumulation of lactate and a concurrent reduction in secondary bile acids [57]. The observed surge in *Lactobacillus* may reflect an ecological adaptation to altered gut chemistry and barrier status in taurine-deficient dogs.

Similarly, the enrichment of *Streptococcus*, a facultative anaerobe often considered an opportunistic taxon, suggests microbial dysbiosis or ecological drift within the gut of taurine-deficient individuals. In canine chronic enteropathies, *Streptococcus* species are consistently associated with inflammation and disrupted microbial communities [57,59]. These bacteria are not only capable of thriving in dysbiotic conditions, but also express BSH enzymes, enabling them to persist in bile-rich environments [58]. Their increased presence may therefore result from both reduced colonization resistance and changes in bile acid metabolism, creating a niche in which bile-tolerant, mucosa-associated taxa such as *Streptococcus* can expand.

Conversely, within the same phylum (*Firmicutes*), a notable decrease in the class *Negativicutes* was observed in the VL group. Although *Negativicutes* class represent a minor component of the canine gut microbiota, they include families which are well-known producers of short-chain fatty acids (SCFAs), including acetate and propionate [60]. These metabolites are crucial for maintaining colonic epithelial integrity, fueling colonocyte metabolism, and modulating mucosal immune responses [60,61]. In canine models, a reduction in *Negativicutes* and their associated SCFAs production has been previously linked to chronic enteropathies and gut dysbiosis [61]. A reduction in these SCFAs-producing taxa may therefore signal impaired colonic fermentation and reduced trophic support for epithelial cells, and thus a potentially weakened barrier integrity in taurine-deficient dogs.

Interestingly, a trend toward reduced abundance of *Gammaproteobacteria* was observed in the L group. Although this class of facultative anaerobic bacteria is frequently associated with dysbiosis and inflammatory conditions, owing to their ability to thrive in inflamed and oxygen-rich niches, they also include commensal species that contribute to normal gut function and microbial diversity [62,63,64]. Therefore, their decline might suggest a transition toward a more anaerobic intestinal environment or reflect competitive displacement by other expanding taxa, such as *Lactobacillales*, known to dominate in certain nutrient or redox conditions.

Finally, a significant increase in the genus *UCG-004* (Family *Erysipelatoclostridiaceae*, Order *Erysipelotrichales*) was observed in taurine-deficient dogs (L group) compared to controls. Although typically a minor component of the gut microbiota, members of the *Erysipelotrichaceae* family have been linked in mice to diets rich in fat, albeit without corresponding improvements in lipid digestibility in the canines [65,66]. In dogs, taxa within this family have been associated with carbohydrate metabolism and short-chain fatty acids production, as well as with reduced markers of protein metabolism in certain contexts [66,67]. While some *Erysipelotrichaceae* genera have been implicated in inflammation-related intestinal and metabolic disorders in humans, others appear to be enriched in healthy dogs compared to those affected by acute hemorrhagic diarrhea or inflammatory bowel disease [68,69]. However, to date, no published studies have specifically investigated the role of *UCG-004* in the canine gut microbiome, and its functional implications remain largely unknown. This genus may represent a sensitive microbial responder to altered intestinal conditions associated with taurine deficiency, but further investigation through targeted metagenomic and metabolomic analyses is required.

These findings could suggest a potential interplay between taurine deficiency and gut microbiota alterations, particularly affecting mucosa-associated and bile-sensitive taxa. However, the clinical interpretation of taurine status remains uncertain, as universally accepted and breed-specific reference intervals are not currently available. Although all dogs underwent comprehensive clinical and cardiac evaluations confirming their health status at the time of sampling, reduced taurine levels in this cohort should be considered only as a potential indicator of susceptibility within the exploratory framework of this pilot study, and not as evidence of disease. Moreover, the lack of universally accepted reference ranges for plasma taurine concentrations represents an additional limitation, as it may influence the classification of dogs into normotaurinemic and hypotaurinemic groups. Another limitation of this study is the relatively small number of enrolled dogs, which was due to the stringent inclusion criterion of selecting only individuals consuming the same commercial diet, to minimize dietary variability as a confounding factor. Also, the study population included a higher proportion of females and a wide age range, which may introduce biological variability. Furthermore, all clinical and nutritional assessments were performed by the same veterinarian (DVM), which, while ensuring consistency in measurements, may limit generalizability. Additionally, the study did not include functional analyses such as bile acid profiling or metabolomics, which would help determine whether the microbial compositional differences observed reflect measurable changes in microbial metabolic activity that could influence taurine availability. As a result, interpretation of certain microbial shifts necessarily relies on previously published findings. Taken together, these factors further support the preliminary nature of the present data. Future studies, with a larger number of dogs, combining microbiome and metabolome profiling may help elucidate whether microbial compositional changes also reflect or drive disruptions in taurine-related metabolic pathways.

## 5. Conclusions

This study highlighted that Golden Retrievers may develop hypotaurinemia and hypocobalminemia despite being fed a complete and balanced diet. Notably, dogs with reduced taurine levels showed distinct differences in gut microbiota composition, inclu-ding shifts in taxa associated with bile salt metabolism and onset of gastrointestinal diseases or inflammation, suggesting a potential state of subclinical dysbiosis. These fin-dings indicate a possible association between hypotaurinemia and alterations in the gut microbiota, supporting the hypothesis that microbial imbalances may contribute to im-paired taurine absorption. However, further investigations with larger cohorts and the integration of metabolomic analyses are warranted to clarify whether these microbial alterations also translate into functional metabolic consequences.

## Figures and Tables

**Table 1:** The chemical composition of the diet fed to the dogs.

Analytical Constituent	As Fed	Dry Matter
Crude protein	25%	27.17%
Crude fat	16%	17.39%
Crude fiber	3%	3.26%
Crude ash	7%	7.61%
Calcium	1.7%	1.85%
Phosphorus	1.05%	1.14%
Metabolizable energy	3840 Kcal/kg	-

**Table 2:** The nutritional statuses of the enrolled dogs. Body weight is expressed in kilograms (kg), BCS is based on a 9-point scale, and MCS is evaluated using a 4-point scale.

Subject ID	Weight (kg)	BCS	MCS
1	29	6	4
2	23	6	4
3	25.5	6	4
4	21	5	4
5	22.5	6	4
6	27.5	6	4
7	29.2	6	4
8	29.5	6	4
9	22.3	5	4
10	29	7	4
11	33.5	6	4

**Table 3:** Taurine serum concentrations.

Subject ID	Taurine (nmol/mL)
1	71.4 ^1^
2	196.1
3	51.57 ^1^
4	35.49 ^1^
5	33.13 ^1^
6	96.11 ^1^
7	104.8 ^1^
8	79.06 ^1^
9	175.63
10	155.86
11	31.85 ^1^

^1^ Taurine values below reference ranges (110–272 nmol/mL).

**Table 4:** Fecal evaluation.

Subject ID	Consistency	Color	Foreign Material
1	4	Brown	None
2	4	Brown	None
3	5	Light Brown	None
4	6	Brown	None
5	4	Brown	None
6	4	Brown	None
7	4	Brown	None
8	5	Brown	None
9	4	Brown	None
10	4	Brown	None
11	4	Brown	None

## Data Availability

The original contributions presented in this study are included in the article/Supplementary Material. Further inquiries can be directed to the corresponding authors.

## Supplementary Materials

The following supporting information can be downloaded at https://www.mdpi.com/article/10.3390/vetsci12121120/s1, File S1: serum parameters concentrations; File S2: ZIP archive of CSV files containing statistical analyses of gut microbiota composition, including alpha and beta diversity and taxa abundances.

## Abbreviations

The following abbreviations are used in this manuscript:

DCM	Dilated Cardiomyopathy
TMAO	Trimethylamine N-Oxide
CBC	Complete Blood Count
BCS	Body Condition Score
MCS	Muscle Condition Score
DVM	Doctor of Veterinary Medicine
PCoA	Principal Coordinates Analysis
ANOSIM	Analysis of Similarities
PERMANOVA	Permutational Multivariate Analysis of Variance
ANCOM	Analysis of Compositions of Microbiomes
ANCOM-BC	Analysis of Compositions of Microbiomes with Bias Correction
FDR	False Discovery Rate
SCFAs	Short-Chain Fatty Acids
